# 1 < 2 and 2 < 3: Non-Linguistic Appreciations of Numerical Order

**DOI:** 10.3389/fpsyg.2013.00005

**Published:** 2013-01-25

**Authors:** Ursula S. Anderson, Sara Cordes

**Affiliations:** ^1^Department of Psychology, Boston CollegeChestnut Hill, MA, USA

**Keywords:** ordinality, numerical cognition, quantity, non-verbal, infants, non-human animals

## Abstract

Ordinal understanding is involved in understanding social hierarchies, series of actions, and everyday events. Moreover, an appreciation of numerical order is critical to understanding number at a highly abstract, conceptual level. In this paper, we review findings concerning the development and expression of ordinal numerical knowledge in preverbal human infants in light of literature about the same cognitive abilities in non-human animals. We attempt to reconcile seemingly contradictory evidence, provide new directions for prospective research, and evaluate the shared basis of ordinal knowledge among non-verbal organisms. Our review of the research leads us to conclude that both infants and non-human animals are adapted to respond to monotonic progressions in numerical order, consonant with mathematical definitions of numerical order. Further, we suggest that patterns in the way that infants and non-human animals process numerical order can be accounted for by changes across development, the conditions under which representations are generated, or both.

## Introduction

Mathematicians Frege (1879/1967) and Russell (1903/1996) defined number as the class of all classes that shows a one-to-one correspondence with a given class in an attempt to cast number in terms of logic. Piaget (1941/1965) described number as the property of a set that remains invariant when other perceptual characteristics (e.g., color, size, and density) of the set change. What both definitions mean is that “oneness” is the class of all singletons, “twoness” the class of all doubles, and so forth. For example, “threeness” characterizes the number of sides of a triangle, leaves of a shamrock, and notes in a musical triplet. Describing number in this way indicates that number is an abstraction – a characteristic based on a *single* property of stimuli independent of other properties – that conceptualizes a collection of discrete things.

The aforementioned definitions of number rest on the observation that the natural world is filled with things that exist in aggregates, collections, or sets (Mill, 1859; Conant, 1896). Cardinality answers questions about “how many” things are in a collection, illustrating a set’s size. We know that infants as young as 6 months of age appreciate differences in the size of large sets when there is at least 1:2 magnitude of difference between sets (e.g., 4 vs. 8, 8 vs. 16, 7 vs. 21, and 16 vs. 32; Xu and Spelke, 2000; Lipton and Spelke, 2003, 2004; Xu, 2003; Wood and Spelke, 2005; Xu et al., 2005; Cordes and Brannon, 2008a). Additionally, infants appreciate differences in the size of small sets (e.g., 1 vs. 2 and 2 vs. 3), but in contrast to large set discriminations, their ability to discriminate changes in the cardinality of small sets does not appear to be ratio-dependent (Feigenson, 2005; Kobayashi et al., 2005; Cordes and Brannon, 2009b). Furthermore, knowledge about numerical cardinality is not restricted to humans. Non-human animals like primates, birds, and fish discriminate between sets based on the number of things that each collection contains (Emmerton, 1998; Jordan and Brannon, 2006b; Jordan et al., 2008b; Tomonaga, 2008; Merritt et al., 2009; Agrillo et al., 2010).

Collections possess only one size, but they can be arranged in a variety of ways. The idea of arranging collections such that relations of order stand between the cardinality of sets concerns ordinality^1^. Ordinality answers questions about “which one” the set is relative to other sets. To recognize numerical ordinal relations, an organism first must detect differences between the cardinality of sets. Thus, cardinal and ordinal understandings about number are intertwined. The ability to discriminate numerical cardinality, however, does not imply an ordinal understanding of number. In other words, being able to determine that various sets contain a different number of things does not mean that one knows that one set contains more or less items than another set.

Ordinality is an important aspect of numerical cognition. For one, it concerns mathematical ideas. In mathematics, the inequalities “greater than” and “less than” are example relations possessing the four properties that must hold for order to exist^2^. The formal counting system (counting 1, 2, 3, 4, etc.) is based on ordering successive sets of things so that any set paired with its nearest neighbor leaves one member left over (Stevens, 1951). Furthermore, the ability to numerically order sets of things is a basic skill linked to the mathematical skills of preschoolers, adolescents, and adults (Halberda et al., 2008; Libertus et al., 2011; Lyons and Beilock, 2011). Ordinality is also important because a fully developed concept of number involves integrated cardinal and ordinal knowledge that holds across sensory modalities and research methods and allows organisms to perform math-like operations. The aforementioned idea comes from the various ways that researchers attempt to define a non-verbal concept of number. Piaget (1941/1965) characterized the development of a number concept as the synthesis of a child’s understanding about the cardinal and ordinal aspects of number. Using the language of mathematics, Gallistel, 1989, 1993 and Gallistel and Gelman (1992) wrote that a concept of number is demonstrated when one can perform operations that are isomorphic to arithmetic and mathematical relations (e.g., >, <, =, and ≠). Other authors focus on transfer of learning across modalities and methods (Davis and Pérusse, 1988; Dehaene, 1997). For these reasons, investigating the extent to which non-verbal organisms, both human infants and non-human animals, appreciate numerical order is important for assessing the richness of their conceptual knowledge about number.

The goal of this paper is to integrate what we know about how preverbal human infants process and represent numerical order with literature about the same abilities in non-human animals^3^.

Our review revealed that both infants and non-human animals are adapted to respond to monotonic progressions in numerical order and positive mappings between number and other quantities. There are, however, notable differences in the way that human and non-human species process numerical order. These differences could be accounted for by development, the conditions under which representations were generated, or both of the aforementioned. Our comparative examination among non-verbal organisms provides information about when an appreciation of numerical order emerged in the phylogenetic scale, how it increases in complexity with development, and the extent to which it is independent of language.

## Non-Verbal Systems of Quantification

Before reviewing the literature, it is important to characterize the cognitive mechanisms putatively responsible for processing and representing the cardinality. Evidence from both non-human animals and humans throughout the life span supports the existence of two distinct systems for representing quantity: an analog-magnitude system and an object-based individuation system. Given it would be impossible to identify order among quantities without the ability to track cardinality, one or both systems are necessarily involved in the processing of numerical order. In reviewing the literature, we aim to take a comparative approach to determining the level of involvement of these two systems in developmentally and evolutionarily early appreciations of numerical ordering.

The most prominent system for representing discrete quantity is the analog-magnitude one. The analog-magnitude mechanism supports continuity in the mode of processing because the system handles both large and small values (Gallistel and Gelman, 2000; Cordes et al., 2001). In analog-magnitude systems, discrete quantities are encoded as continuous noisy magnitudes such that an accumulator fills up in nearly equal increments for each entity counted (Gibbon, 1977; Meck and Church, 1983; Church and Meck, 1984; Gibbon and Meck, 1984). There is a scalar property to the noise in the accumulator such that variability in how much the accumulator fills up increases proportionally to the mean value in the accumulator. This results in discriminations becoming less precise as the quantity increases. When detecting differences between quantities, the measure of closeness between a current value and a value stored in memory is the ratio between the values.

An accumulator with scalar noise coupled with ratio-based comparisons is consonant with the observation that quantity discriminations obey Weber’s law. Specifically, that the discriminability of two objective values is dependent up on their ratio, not their absolute difference. In particular, if values are encoded and processed as noisy magnitudes then (a) the closer two values are, the harder it is (and longer it takes) to determine which is the larger or smaller one and (b) the larger two values are, the harder it is (and longer it takes) to determine which is the larger or smaller one. These response patterns have been named the numerical distance and size effects. Together the aforementioned effects create the numerical ratio effect, the finding that discrimination ability declines (and response latency increases) when the numerical ratio between compared values approach a value of one (Dehaene et al., 1998). In summary, the analog-magnitude system generates fuzzy, approximate representations of both small and large quantities.

The second system proposed to account for how non-verbal organisms processes and represent quantitative information is based on precise or exact individuation of objects (Simon, 1997; Leslie et al., 1998; Carey and Xu, 2001; Hyde and Wood, 2011). Object-based individuation mechanisms draw upon theories of visual object attention (FINST mechanism, Pylyshyn, 1989; object-file model, Kahneman et al., 1992) and parallel individuation and working memory storage for objects (Vogel et al., 2001; Feigenson, 2008). The idea is that temporary placeholders (object-files, indexes, or unique mental symbols) are assigned in parallel to each relevant object perceived by the visual system when organisms scan an array. Object-based individuation systems attempt to place currently perceived placeholders in one-to-one correspondence with the placeholders from preceding scenes that are in working memory.

In general, infants can hold an exact representation of no more than three items in working memory. This limit is based on evidence that infants can discriminate between small sets (Jordan and Brannon, 2006a), but not discriminate small from large sets (Xu, 2003; see also Cordes and Brannon, 2008b; Cordes and Brannon, 2009a). For example, infants can resolve manual search problems with no more than three items. When 14-month-olds watch an experimenter place three balls in an opaque box and then remove two balls, they search for the third ball in the box. When four balls are placed in the box and two balls are removed, infants do not search for balls remaining in the box (Feigenson and Carey, 2003; Feigenson and Halberda, 2004). In contrast, the limit for non-human animals and human adults is slightly higher. Based on empirical evidence about the limits of parallel individualization and working memory capacity, human adults and non-human animals can hold an exact representation of four or five items in working memory (Feigenson, 2008; Wood et al., 2008). Object-based individuation systems, thus, support discontinuity in the mode of quantitative processing. The number of objects that can be simultaneously tracked or held in working memory is limited so the processing of large values must be left to a secondary system (i.e., the analog-magnitude system).

## Ordinal Basis of Relative Quantity Judgment

Humans and non-human animals have access to two distinct systems for representing cardinality, but how do they make ordinal judgments about these representations? Humans and non-human animals encounter situations in their daily lives to which the use of relative quantitative information would be advantageous (Gallistel, 1989; Hauser, 1997; Tomasello and Call, 1997; Wynn, 1998). For example, infants may reach for the hand that contains the greater number of cereal bits and animals may engage in aggressive interactions only with conspecifics that possess a fewer number of allies than they do. Number, though, tends to vary with other continuous quantities (e.g., surface area, density, volume, brightness, inter-item distance, perimeter/contour length, etc.) in systematic ways. For example, the amount of exposed Cheerio surface increases as the number of Cheerios in your father’s hand increases. Because a variety of quantitative information is available in situations like these, an organism’s decisions may not be based solely on number.

Number is naturally so tightly interwoven with other quantitative properties that it is difficult for researchers to design experimental tasks that isolate number. Early contributions to the literature, thus, focused on describing how preverbal human infants and non-human animals processed ordinal relations about quantitative information without specifically isolating number’s contribution using relative quantity judgments (RQJ). RQJ tasks rest on an organism’s natural tendency to choose the greatest amount of desirable things (or choose the least of undesirable things) if they are capable of distinguishing between unequal quantities. RQJ is described as the simplest quantitative skill because it does not require the comprehension of precise or absolute number (Davis and Pérusse, 1988). This means that reliably choosing the most of something does not imply knowledge about how divergent the collections are. RQJs are still an important ordinal skill because they give animals the means to maximize food intake (Davis, 1993). For these reasons, we look to the literature about RQJ with discrete quantities to provide insight about the shared basis of processing and representing ordinal relations that involve number.

### Relative quantity judgment in infants

Unlike non-human animals, human infants are not forced to forage for food for survival. Thus, very few studies have used RQJs to investigate ordinal understandings in human infants. Evidence from these studies, however, reveals that infants are capable of making active responses to determine which of two locations contains the greater amount of desirable items, whether they be toys or food. These ordinal abilities, however, appear to be dictated by the cardinal values of the sets under question.

An early experiment involving RQJ in infants showed that 14-month-olds were able to identify the larger of two small sets of non-visible discrete quantities (Sophian and Adams, 1987). Two sets of toys (1 vs. 2) were shown to infants and then covered with transparent boxes. Infants were then allowed to reach for the box they desired. If the smaller set was chosen, contact with that set was prevented and the infant was verbally encouraged to select the larger set. The procedure was nearly identical during testing except that opaque boxes were used to cover the two sets of toys and insertion-deletion transformation problems (e.g., 1 vs. 1 + 1; and 3 vs. 2 − 1) were also presented. Infants selected the set with the most toys more often than chance, which demonstrated infants were capable of making a judgment about the ordinal relation between two small quantities, at least after training.

Two more recent studies demonstrated that infants spontaneously make RQJs between sequentially presented, non-visible amounts as early as 10-months of age (Feigenson et al., 2002; Feigenson and Carey, 2005). Ten- and 12-month-olds were shown two sets of crackers that were sequentially placed into two opaque buckets. The infants were then allowed to crawl toward the bucket that they desired on a single-trial. By only presenting a single-trial, researchers were able to evaluate spontaneous ordinal judgments in which training played no part. Both 10- and 12-month-old infants chose the set with the most crackers for comparisons that were defined by small quantities (1 vs. 2 and 2 vs. 3), but not for comparisons in which one set was small and the other large (1 vs. 4, 2 vs. 4, 3 vs. 4, and 3 vs. 6). This was the case even though the ratio between quantities was the same for certain small and large quantity comparisons (e.g., for a 1:2 ratio: success with 1 vs. 2, but failure with 2 vs. 4 and 3 vs. 6). Successful ordinal comparison of small sets (<4 items) and failure when sets span the small/large size divide suggests that the infants relied on an object-based representation system that was capable of storing up to three items. When ordinal comparisons crossed the small/large boundary, incompatibility between representations of small (via object-based individuation) and large sets (via analog-magnitude) resulted in a failure to choose the larger set.

It is important to note that there is also evidence that suggests that infants use analog-magnitude representation when making judgments about ordinal relations. Using procedures similar to Feigenson et al. (2002), a study found that 10- to 12-month-olds were equally successful at choosing the larger amount of discrete food items with a set that was below (1 vs. 2) and above (5 vs. 10) the capacity limit predicted by object-based individuation models (Van Marle and Wynn, 2011). This result suggests that an approximate representation system is at work when responding to quantitative order. Because only one large comparison was presented, we do not know if RQJs involving large sets display a ratio signature during infancy. Additional experiments are needed to provide information about the conditions under which preverbal infants use analog-magnitudes when making decisions about the ordinal relations that stand between large quantities.

In sum, both object-based and analog-magnitude representations appear to play a role in RQJs during infancy. When choosing between two small sets, like 1 vs. 2 pieces of cereal, or choosing between two large sets, like 5 vs. 10 pieces of cereal, infants as young as 10-months of age successfully reach for, and crawl toward, the largest discrete amount. When ordinal comparisons cross the small/large set size boundary, like choosing between 1 vs. 4 pieces of cereal, incompatibility between representations generated via object-based individuation and analog-magnitude systems results in infants failing to choose the larger discrete amount.

### Relative quantity judgment in non-human animals

A similar small/large set size distinction holds with the spontaneous judgments that non-human animals make about the ordinal relations between quantities. Set size limits in keeping with a limited capacity, exact object-based individuation system were reported in rhesus monkeys (Hauser et al., 2000), salamanders (Uller et al., 2003), and horses (Uller and Lewis, 2009) who chose between two unequal sets of food items. Specifically, even when the ratio between quantity pairs was equivalent across the small/large quantity divide: (a) rhesus monkeys selected the larger of two sets when at least one set contained four or fewer items (e.g., 2 vs. 3, 3 vs. 4), but failed otherwise (e.g., 4 vs. 6, 4 vs. 8); (b) salamanders selected the larger of two sets when both sets contained three or fewer items (1 vs. 2 and 2 vs. 3), but failed otherwise (3 vs. 4 and 4 vs. 6); and (c) horses selected the larger of two sets when both sets contained three or fewer items (1 vs. 2 and 2 vs. 3), but failed otherwise (4 vs. 6)^4^. Together, these findings point to the conclusion that non-human animals can store and represent from three to four objects when making ordinal decisions about quantities.

Furthermore, the existence of a phylogenetically shared analog-magnitude system for processing and representing ordinal relations is supported by many studies. When apes and monkeys are allowed to choose between sets of unequal discrete food and non-food items, their responses are dependent on the ratio between quantities (Call, 2000; Beran and Beran, 2004; Anderson et al., 2005, 2007; Suda and Call, 2005; VanMarle et al., 2006; Beran, 2007; Hanus and Call, 2007; Stevens et al., 2007; Addessi et al., 2008; Beran et al., 2008a,b; Tomonaga, 2008; Evans et al., 2009; Schmitt and Fischer, 2011).

Evidence of ratio-dependent RQJs is not limited to non-human primates. African elephants (Perdue et al., 2012), crows and African gray parrots (Zorina and Smirnova, 1996; Al Ain et al., 2009; Bogale et al., 2011), coyotes and dogs (Ward and Smuts, 2007; Baker et al., 2011), bears (Vonk and Beran, 2012), sea lions (Abramson et al., 2011), salamanders (Krusche et al., 2010), and swordtail fish (Buckingham et al., 2007) show ratio-dependent ordinal judgments consistent with the predictions of analog-magnitude representation^5^. It is important to note that a set size signature was not found in these studies. This was the case even though some subjects did not receive an extensive number of training or test trials (Ward and Smuts, 2007; Krusche et al., 2010; Baker et al., 2011) and some subjects were experimentally naïve (Anderson et al., 2005, 2007; Buckingham et al., 2007; Ward and Smuts, 2007; Krusche et al., 2010; Abramson et al., 2011; Bogale et al., 2011).

Additional evidence supporting the involvement of analog-magnitudes in ordinal quantity judgments is found when non-human animals make RQJs with non-visual sets. Male beetles choose to spend more time inspecting substrates on which the most female beetles had been located, but only when there was at least a three-fold ratio of difference between compared sets (i.e., succeeded with 1 vs. 3 and 1 vs. 4; failed with 2 vs. 4 and 1 vs. 2; Carazo et al., 2009). In addition, a non-human primate selected the largest quantity after hearing discrete food items dropped into two opaque containers (all pairs from 1 to 5) even when item presentation time was unconfounded with quantity. The subject’s responses were affected by the ratio between quantities when making auditory RQJs (Beran, 2012)^6^. Overall, these tasks reveal ratio-dependence, even for small sets, which is indicative of the modality-independence of ordinal understanding^7^.

Similar to the findings from RQJs with human infants, a non-human animal study (Hunt et al., 2008) indirectly suggests that two core representation systems are at play during RQJs. Consonant with the predictions of object-based individuation, robins chose the larger of two sets of food items when both sets contained four or fewer items (1 vs. 2, 2 vs. 3, and 3 vs. 4), but not when both sets contained four or more items (4 vs. 5, 4 vs. 6, 6 vs. 8, and 8 vs. 10) even though the ratio between some quantity pairs was equivalent across the small/large quantity divide. On the other hand, the robins successfully chose the larger set for one quantity pair that had a large ratio of difference (i.e., 1:2 ratio between 4 vs. 8), which is suggestive of an analog-magnitude system at work. Thus, songbirds respond to ordinal relations when both sets contain small quantities or when there is a 1:2 ratio of difference between large quantities. Because only one large comparison was presented, we cannot say that the ordinal responses of robins showed a ratio signature, though.

More compelling evidence in support of the two-system view is provided in recent empirical work investigating the social judgments of gregarious fish that prefer to join large rather than small groups (Bisazza et al., 2010; Agrillo et al., 2012). When guppies and mosquitofish made RQJs between two large shoals (>3 members), choices were dependent upon the ratio between shoal sizes. In contrast, when both shoals contained four or fewer members (1 vs. 2, 1 vs. 3, 1 vs. 4, 2 vs. 3, and 3 vs. 4), their choices were more accurate and consistent with object-based representation (Bisazza et al., 2010; Agrillo et al., 2012). Notably, the ability to appreciate ordinal relations with analog-magnitude representation showed developmental progression in guppies (Bisazza et al., 2010). Newborn guppies chose to spend more time near the larger of two small shoals, which illustrates that the exact, object-based representation system is at work at birth. Their ability to make RQJs between larger shoals (>4 members) emerged with increasing age and social experience, suggesting that analog-magnitudes are less salient early in development.

In sum, these findings illustrate that the ordination of quantities is modality-independent and extends across animal taxa. Both core representation systems are involved when non-human animals make ordinal judgments. When non-human animals are given a single opportunity to select the larger of two desirable discrete quantities, they do so by creating: (a) exact object-based representations if both sets are small, (b) approximate analog-magnitudes if both sets are large, and (c) both kinds of representations if one set is small and the other is large, which results in incompatibility. When making repeated ordinal judgments about quantities, non-human animals are largely dependent on analog-magnitude representation of both small and large discrete quantities. In other words, non-human animals show ratio-dependent responses without a set size signature when making repeated ordinal judgments about quantities.

### Conclusions about relative quantity judgment

When it is advantageous to choose the larger set of discrete things, infants and non-human animals do so, within the limits of their representation systems. Infants and non-human animals travel to, and reach for, the largest collection of desirable items, whether they be food, toys, or social partners. Some animals are even able to do so solely from the sound of items dropping into containers. Ordinal choices occur via an approximate analog-magnitude representation when the ratio between compared sets is large enough and via an exact object-based representation system when the sets being compared are both small. Furthermore, the ordinal quantity judgments of both infants and non-human animals indicate an interaction between these two core systems of representation.

Evidence of both systems at work is most prominent in single-trial investigations of spontaneous ordinal judgments in both human infants and non-human animals. Under this constraint, infants and non-human animals reveal ordinal competence when both to-be-compared sets are small or both are large (provided a favorable ratio). In contrast, when ordinal comparisons cross the small/large size boundary, incompatibility between representations of small (via object-based individuation) and large sets (via analog-magnitude) results in a failure to choose the larger quantity. This pattern mirrors how infants discriminate cardinal number. In particular, young infants can tell that two numerical sets are different in size when both sets are small or when both sets are large (Jordan and Brannon, 2006a; Cordes and Brannon, 2009a), but fail to do so when one set is small and the other is large (e.g., 2 vs. 4; Xu, 2003; Wood and Spelke, 2005; Cordes and Brannon, 2009a).

The ordinal quantity judgments of infants show a three-item limit for object-based representation, which is consistent with the literature about the detection of differences in the numerical size of sets during infancy. Non-human animals, even those that are newly born (Bisazza et al., 2010), show a more flexible three- to four-item limit when making RQJs. Note that the limit for adult humans is closer to no more than four or five objects for exact, object-based individuation (Feigenson, 2008; Wood et al., 2008). Together, this information reveals a dichotomy in the development of the exact object-based representation system across human and non-human animals. Humans experience expansions in working memory from infancy to adulthood, which co-occur with an increase in the capacity limit of the object-based representation system. In contrast, one study (Bisazza et al., 2010) suggests that non-human animals are endowed with a larger, but fixed-capacity object-based individuation system at the time of birth. Future research should investigate if non-human animals experience similar developmental expansions in their working memory or if their object-based representations are fixed throughout development.

Even so, at least one non-human species shows developmental advancement in the analog-magnitude system (Bisazza et al., 2010). The findings showed that newly born guppies are less likely (or not able) to use analog-magnitudes when deciding to affiliate with the larger of two shoals. This phenomenon mirrors the increase in precision seen in analog-magnitude representations across human development. The ratio that must exist for humans to discriminate that the size of two large sets differs decreases from a threefold magnitude of difference (e.g., 4 vs. 12) at birth to a twofold magnitude of difference (e.g., 4 vs. 8) at 6 months of age to a 1.5 ratio (e.g., 4 vs. 6) around 10 months of age to a 1.14 ratio (7 vs. 8) in adulthood (Xu and Spelke, 2000; Lipton and Spelke, 2003, 2004; Xu, 2003; Wood and Spelke, 2005; Xu et al., 2005; Xu and Arriaga, 2007; Cordes and Brannon, 2008a; Halberda and Feigenson, 2008; Izard et al., 2009).

When given repeated trials, though, the RQJs of non-human animals are almost entirely ratio-dependent. This pattern points to an analog-magnitude base for representing discrete quantity when given more than a single opportunity make choices. In this case, analog-magnitude representation holds across the small/large set size divide even for experimentally naïve subjects given a low number of reinforced trials. For example, experimentally naïve dogs were presented with eight pairs of discrete quantities (1 vs. 4, 1 vs. 3, 2 vs. 5, 1 vs. 2, 2 vs. 4, 3 vs. 5, 2 vs. 3, and 3 vs. 4) only once, but still their responses showed a ratio signature (Ward and Smuts, 2007). Further, analog-magnitude representation when making RQJs mirrors the pattern found when non-human animals discriminate that two numerical sets are the same (or different) in size (Boysen and Berntson, 1989; Emmerton, 1998; Jordan and Brannon, 2006b; Jordan et al., 2008a; Tomonaga, 2008; Merritt et al., 2009; Agrillo et al., 2010). The parallel that exists between cardination and ordination means that repeated assessments about whether two quantities are different or the same in size are governed by the same representation systems as repeated assessments about whether one numerical set is larger or smaller than another set. Unfortunately, only one infant study has broached the topic of large set RQJs (Van Marle and Wynn, 2011) so it remains to be seen whether the same pattern holds for our youngest counterparts.

In sum, we can conclude that ordinal quantity judgments are an evolutionarily ancient, developmentally early, non-linguistic capacity in the phylum Chordata that spans a species’ social system and ecological niche. Although providing evidence of primitive ordinal understanding in non-verbal organisms, it should be noted that the RQJ paradigm fails to disentangle number from other quantitative information like surface area, presentation time, volume, and hedonic value^8^. For this reason, it is unclear whether infants and non-human animals in these studies relied upon number, continuous quantity, or both number and continuous quantity when making their judgments. In fact, follow-up experiments to these studies and control trials within some studies suggest that non-numerical quantities dictated responding in some infant (Feigenson et al., 2002) and non-human animal studies (Zorina and Smirnova, 1996; Stevens et al., 2007; Tomonaga, 2008; Krusche et al., 2010; Vonk and Beran, 2012; but, see, Hauser et al., 2000; Beran, 2007; Bogale et al., 2011). As such, these studies provide indirect evidence suggestive of evolutionarily and developmentally early appreciations of number-related ordinal relations. They do not, however, distinguish number as the driving force behind successful ordinal judgments. If and under what conditions preverbal human infants and non-human animals store and compare exact object-files, approximate analog-magnitudes, or both kinds of representations about numerical information in the absence of covarying cues from continuous quantity is the matter that we consider in the next sections.

## Understanding Numerical Order in the Absence of Non-Numerical Cues

Why do scientists attempt to understand the unique influence of number on the behavior of non-verbal organisms, particularly if number varies systematically and reliably with other quantitative properties (e.g., as the number of my allies increases so does the overall loudness of their vocalizations)? Some researchers argue that the origins of quantitative competence are rooted in discrete number, whereas, others argue for non-numerical origins in which initial representations are amount-based (for reviews, see, Mix et al., 2002; Henik et al., 2012). For this reason, attempts must be made to capture the potential differences that exist when non-verbal organisms order sets of things based on number and when they order using a host of non-numerical quantitative cues. Surprisingly, developmental investigations of this sort have provided a range of conflicting results making it unclear whether or when infants are truly capable of understanding numerical order unconfounded with continuous quantities. In contrast, non-human animal studies reveal a robust pattern of successful ordination. In this review, we attempt to shed light on the apparent inconsistencies within and between the two bodies of literature.

Ordinal numerical knowledge in preverbal infants is typically assessed via looking-time measures in which infants are exposed to numerical sequences. Infants are habituated to sequentially presented sets of items that illustrate an ordinal direction (e.g., 1  → 2 → 3, if the arrow denotes “comes before”) and then tested with novel sequences that illustrated a reversal in ordinal direction (e.g., 6 → 5 → 4). Infants can be said to recognize order among numerical sets if they look longer at sequences that do not obey the ordinal rule that they viewed during habituation or familiarization. To study how non-human organisms process and represent numerical order, researchers turned to mathematical definitions of an order relation (Green and Stromer, 1993). In this paradigm, sequential responses that illustrate a direction of order are established (e.g., 1 → 2 → 3, if the arrow denotes “responded to before”). If organisms apply the learned sequential response to numerically novel sequences (e.g., 4 → 5 → 6) in the absence of reinforcement then the inference is that they appreciate ordinality.

Regardless of experimental paradigm, this line of research necessarily prevents continuous quantity from influencing an organism’s responses so that number’s unique contribution can be evaluated. In the experiments we review, the effects of cumulative area, item perimeter or contour length, and array density or inter-item distance are controlled (except where otherwise noted). Thus, the sets that infants and non-human animals view are numerical sets for which cardinality is the only property relevant to be discriminated, and the order that exists among these sets is a function of progressions in cardinality^9^.

### Ordinal numerical knowledge in infancy

There are only a handful of studies looking at ordinal numerical knowledge in infancy. This is likely because the earliest investigations did not show ordinal competence in infants. Cooper (1984) reported that 10- to 12-month-old infants did not detect ordinal relations between quantity sequences in which number was confounded with other quantitative properties (e.g., surface area). Infants habituated with ascending (1 → 2; 2 → 3; and 3 → 4) or descending (4 → 3; 3 → 2; and 2 → 1) two-set sequences failed to dishabituate to reversed order novel test sequences even though continuous extent was confounded with number. Similarly, Strauss and Curtis (1984) reported that 16- to 18-month-olds did not recognize ordinal relations between two simultaneously presented numerical sets. Using a simple discrimination procedure, infants were reinforced for selecting the larger or smaller number of dots with a single training pair (1 vs. 2, 2 vs. 4, or 3 vs. 4). Then, transfer of the learned ordinal response was assessed by presenting the infants with two numerically novel pairings (pairs from 1 to 5). Although most infants learned to select the larger or smaller numerical set with the original training pair, only a minority of infants continued to make the correct ordinal response with novel numerical pairings. These failures suggested that infants as old as 1.5 years of age did not appreciate ordinal relations.

Given the lack of promise, very little work was done in this area of inquiry for nearly 20 years until the work of Brannon (2002) and Suanda et al. (2008), which capitalized on new knowledge about the ratio-dependency of cardinality discrimination during infancy. In their studies, 9- and 11-month-old infants were habituated to three-set numerical sequences presented sequentially in monotonically ascending (1 → 2 → 4; 2 → 4 → 8; and 4 → 8 → 16) or descending order (16 → 8 → 4; 8 → 4 → 2; and 4 → 2 → 1). Once habituated, a numerically novel ascending and descending sequence was presented (3 → 6 → 12 and 12 → 6 → 3) to test whether a monotonic reversal of ordinal direction would be detected. The 11-month-olds looked longer at, and dishabituated to, the reversed order test sequence. That is, if they were habituated with ascending sequences, they looked longer at the descending sequence during the test phase and vice versa (Brannon, 2002; Suanda et al., 2008). This was the case even when sequences were modified to ensure that absolute set sizes did not serve as a reliable cue for discrimination (Suanda et al., 2008). In contrast, the 9-month-olds looked equally long at ascending and descending test sequences, revealing no evidence of an ordinal appreciation. Based on these findings, the authors concluded that an understanding of numerical order develops between 9 and 11 months of age^10^.

Recent experiments, however, indicate that ordinal number understanding may be present earlier than the 9- to 11-month age range that was initially reported. Using methods similar to Brannon (2002), infants as young as 7-months of age detected changes in ordinal relations when sequences contained only large values (Picozzi et al., 2010). When habituated to numerical sequences that ascended (6 → 12 → 24; 9 → 18 → 36; and 12 → 24 → 48) or descended (48 → 24 → 12; 36 → 18 → 9; and 24 → 12 → 6), 7-month-old infants looked longer at novel numerical sequences that illustrated a reversed monotonic ordinal direction compared to a non-reversed one (4 → 8 → 16 and 16 → 8 → 4). In contrast to Brannon (2002), all sequences contained only large numerical values instead of a mixture of large and small values. Similarly, a study of cross-dimensional transfer of ordinal understanding also provides evidence to suggest that infants appreciate numerical ordinality between large sets earlier than 11-months of age (de Hevia and Spelke, 2010; Lourenco and Longo, 2010). Eight-month-old infants were habituated to a five-set numerical sequence that monotonically ascended (4 → 8 → 16 → 32 → 64) or descended (64 → 32 → 16 → 8 → 4) and then tested with novel five-item *line length* sequences that had a reversed and non-reversed direction of order. Infants looked longer at line length test sequences that illustrated a reversed ordinal direction (de Hevia and Spelke, 2010). Importantly, to distinguish between the ordinal direction of line length sequences during testing, infants needed to encode the ordinal direction of the numerical sequences during habituation.

Why did the 9-month-olds fail to detect ordinal reversals in one study (Brannon, 2002) yet 9- and 7-month-olds succeeded in another study (de Hevia and Spelke, 2010; Picozzi et al., 2010)? Set cardinality is the additional factor responsible for this divergent pattern of results. That is, numerical order is processed and represented at a younger age when numerical sequences contain only large values exclusively represented via the analog-magnitude system. In the most recent studies revealing early ordinal competence (de Hevia and Spelke, 2010; Picozzi et al., 2010), infants were presented exclusively with large sets (>3 items). In contrast, small sets were either exclusively presented or mixed in with large sets in those studies in which all age groups (Cooper, 1984; Strauss and Curtis, 1984) or the youngest age groups (Brannon, 2002; Suanda et al., 2008) failed to detect a reversal in numerical order. These findings indicate that representations from the two core systems are incompatible such that infants fail to recognize changes in ordinal direction when sets within sequences span the small/large set size divide because both core systems are engaged.

Not only are infants tuned to detect cross-dimensional ordinal mappings between number and other quantities, they are also adapted to detect ones that mirror the way number covaries with other quantitative attributes in the natural world. In additional experiments, de Hevia and Spelke (2010) found that 8-month-olds who were familiarized with number-line pairs that illustrated positive ordinal interrelations (smaller set sizes paired with shorter line lengths and larger set sizes paired with longer lines) readily learned these relationships and discriminated between novel stimuli revealing positive (i.e., consistent) and inverse (reverse) pairings of this relationship. In contrast, infants familiarized to inverse ordinal interrelations (small lines paired with large set sizes and vice versa) failed to learn the relationship, showing no difference in the time they looked at positive and inverse ordinal interrelations during the test phase. These experiments provide additional evidence that infants appreciate the ordinal relationships that exist between numerical stimuli, even as young as 8 months provided set sizes are large. But further, these data show that early recognition of ordinality must be consistent with how quantities covary in the real world, suggesting that there is something privileged about the inherent ordering of these quanties, at least in the preverbal mind.

Additionally, there is one more study using a unique design that reveals early detection of numerical ordinality discrimination across small and large sets alike prior to 11 months. In Lourenco and Longo’s (2010) associative learning task, 9-month-olds were habituated to two numerical pairs (2 vs. 4, 3 vs. 6, or 5 vs. 10) for which the relative magnitude of numerical pairs was tied to the color/pattern information of sets. For example, items in the smaller numerical set were always white with black dots, whereas, items in the larger numerical set were always black with white stripes. In the test phase, the numerical pair not presented during habituation was shown with a reversed relative magnitude color/pattern mapping (i.e., items in the smaller set were black with white stripes and items in the larger set were white with black dots). The results revealed that infants looked longer at the reversed number-color/pattern mapping during the test phase. In other words, they expected numerically novel pairs to follow the ordinal rule that they experienced during habituation.

Similarly, a second experiment examining cross-dimensional transfer of ordinal interrelations indicated that young infants process and abstract numerical order across small and large sets (Lourenco and Longo, 2010). Nine-month-olds were habituated to numerical pairs for which there was a systematic mapping between the relative magnitude and color/pattern information. For example, if infants were habituated to a display in which the numerically smaller set was white with black dots (and the larger set was black with white stripes), then during test, holding set size constant, the set with a smaller cumulative area was depicted as white/black dots (consistent ordinal direction) or alternatively, depicted as black/white stripes (reversed ordinal direction). The findings showed that 9-month old infants detected reversals in ordinal direction across the dimensions of space and time. Again, cross-dimensional detection of monotonic reversals in ordinal direction could only occur if infants detected ordinal direction in the numerical sequence. These findings demonstrate ordinal numerical competence in 9-month-olds despite set sizes crossing the small/large set size boundary, which contrasts with the findings of Brannon (2002) and Suanda et al. (2008). We explore one explanation for this discrepancy in the conclusion section.

### Ordinal numerical knowledge in non-human animals^11^

Like human infants, the behavior of non-human primates reveals that they understand ordinal relations among numerical sequences. Rhesus monkeys were trained to touch two-set numerical sequences in ascending (1 → 2; 1 → 3; 1 → 4; 2 → 3; 2 → 4; and 3 → 4) or descending monotonic order (Brannon and Terrace, 1998, 2000; Brannon et al., 2006)^12^. Intermixed with trials of the training sequences were non-differentially reinforced trials of numerically novel test sequences (all possible pairing of 5–9 elements). Monkeys responded in the appropriate order to the numerically novel sequences more often than predicted by chance, which showed that they abstracted ordinal relations. A subsequent experiment showed that learning how to sequentially respond in ascending order to two-set numerical sequences (all possible pairs from 1 to 9 elements) resulted in two of the rhesus monkeys understanding ordinal relations between novel two-set sequences that had values well outside the originally learned range (i.e., all possible pairs of 10, 15, 20, and 30 elements; Cantlon and Brannon, 2006). In contrast, researchers were unable to train a rhesus monkey to sequentially respond in an arbitrary non-monotonic order (3 → 1 → 4 → 2; Brannon and Terrace, 2000), suggesting that responding relied upon the ordinal relationships inherent in the numerical stimuli. Together, findings reveal that rhesus monkeys understand ordinal relations between numerical sets and they can apply their learned ordinal understanding across a wide range of numerical values.

Ordinal understanding of this type is not restricted to the genus *Macaca* or even to non-human primates. Replications with other species reveal that a hamadryas baboon and squirrel monkey (Smith et al., 2003), one of three capuchin monkeys (Judge et al., 2005), and pigeons (Scarf et al., 2011) gain an understanding of ordinal numerical relations from learning to sequentially respond in an ascending manner to numerical sequences. In addition, two bottlenose dolphins gained an understanding about ordinal relations between simultaneously presented numerical pairs (Jaakkola et al., 2005). In particular, they generalized the ordinal rule “choose the least” from training sets (2 vs. 6, 1 vs. 3, 3 vs. 7, 1 vs. 8, 3 vs. 7, 2 vs. 4, and 4 vs. 7) to new pairings (all possible pairs from 1 to 8 items). Together, these findings reveal that ordinal understanding about number spans Old and New world monkeys and the class Mammalia and Aves.

Importantly, the findings indicate that analog-magnitude representations of number governed the ordinal responses of non-human animals. Accuracy and/or response time conformed to Weber’s law for most monkeys, pigeons, and dolphins (Brannon and Terrace, 1998; Smith et al., 2003; Jaakkola et al., 2005; Brannon et al., 2006; Cantlon and Brannon, 2006; Scarf et al., 2011) in support of analog-magnitude numerical representation. In particular, response accuracy declined and response time increased as the ratio between the numerical values being compared approached a value of one. In contrast to the human infant literature, there was no evidence that ordinal knowledge was limited to sets with no more than three or four items, which suggests that object-based representation systems were not involved. There also was no evidence that ordinal knowledge was disrupted by the employment of two distinct systems of representation for number as ratio-dependent responding held across all cardinal values, which implicates the analog-magnitude system. Although some animal subjects were experimentally sophisticated in discriminating between large and small numerical values (Brannon and Terrace, 1998; Brannon et al., 2006; Cantlon and Brannon, 2006), others were not (Smith et al., 2003; Jaakkola et al., 2005; Judge et al., 2005; Scarf et al., 2011). Experimental history, therefore, does not offer a satisfactory explanation for not finding evidence of set size limits. Instead, the findings suggest that non-human animals are less likely to engage an object-based individuation system when tracking numerical order.

### Conclusions about ordinal numerical knowledge

The empirical evidence indicates that both non-human animals and preverbal infants are capable of detecting changes in numerical order. Comparative analysis, however, indicates that the representation systems that infants and non-human animals rely on to detect numerical order differ. Monkeys, birds, and dolphins respond according to numerical order, and the primary representations that they form when doing so are fuzzy, analog-magnitudes. Further, their ordinal responses are dependent on the ratio between numerical sets, not the size of sets. Analog-magnitude representation when responding sequentially to numerical order is in keeping with the representations that non-human animals generate when determining that numerical sets differ in size (Boysen and Berntson, 1989; Emmerton, 1998; Jordan and Brannon, 2006b; Jordan et al., 2008a; Tomonaga, 2008; Merritt et al., 2009; Agrillo et al., 2010). This indicates that the same analog-magnitude system is used to represent numerical information for cardination and ordination; indeed, cardination must occur for ordination to occur.

On the other hand, two core representation systems – an object-based individuation system that processes small sets exactly (<4 items) and an analog-magnitude system that processes large sets approximately (>3 items) – are involved when human infants recognize numerical order. This is illustrated in the finding that infants younger than 11-months of age appear unable to process and represent numerical order with numerical sequences that cross the small/large set size boundary. The same processing incompatibility exists when infants discriminate differences in the number of things that sets contain (Xu, 2003; Wood and Spelke, 2005; Cordes and Brannon, 2009a). This again speaks to the idea that cardination via object-based or analog-magnitude representation must occur for infants to detect order among numerical sets.

We provide two non-mutually exclusive explanations to account for the divergent pattern between human infants and non-human animals. First, the divergent pattern may arise because associative learning paradigms activate a continuous, broadly applicable system for understanding small and large numerical values (i.e., analog-magnitudes). When non-human animals are repeatedly reinforced for touching numerical sets in a progressing order, they rely on analog-magnitude representations to abstract ordinal relationships. On the other hand, when infants are familiarized or habituated to numerical sets (passive viewing conditions), they rely on both core systems. Consistent with the animal literature, infant looking-time patterns did not point to a small/large set size boundary in one study employing associative learning to investigate ordinal knowledge (Lourenco and Longo, 2010). Infants could predict a set’s ordinal class (“larger than” and “smaller than”) from the color and patterning of the elements in sets (e.g., the larger set black rectangles with white dots vs. smaller set white rectangles with black stripes). There was no disruption from activating both core systems – infants succeeded in detecting numerical ordinal relations between small and large sets (e.g., 2 vs. 4 and 3 vs. 6). Although this study does not inform us about the presence of ratio-based responding, at the very least this study suggests that it is easier for infants to construct and compare representations from the two core systems when associative learning is involved.

A second explanation for the divergent pattern of results obtained between infants and non-human animals is a developmental one. The evidence suggests a developmental progression in the ability of infants to process and compare sets when making numerical ordinal judgments. This is illustrated in the finding that infants are not able to process and represent numerical order with sequences that contain both large and small sets until 11-months of age. Although differences across human and non-human species have been investigated, changes across development within non-human animal species have not been thoroughly examined. Therefore it remains to be seen whether this developmental pattern is uniquely human or whether a similar developmental trajectory is mirrored in non-human animals. It is known that guppies show an ontogenetic progression from relying on object-based individuation to relying on both object-based individuation and approximate-magnitude systems when making RQJs (Bisazza et al., 2010). So it may be that non-human animals show a similar progression from greater reliance upon the object individuation system early in development (as has been observed in human infants) to later fluency with integrating representations across the analog-magnitude and parallel individuation systems when number is the only relevant cue. Attempts should be made to investigate developmental patterns in the way that non-human animals understand numerical order to determine if representations of small sets are similarly granted a privileged status early in the development of non-human species (Buhusi and Cordes, 2011).

Despite the differences in the way that infants and non-human animals process and represent numerical order, both preverbal infants and non-verbal organisms understand numerical order in a way that follows the patterns that exist in the natural world. Two sorts of evidence support this claim. First, in nearly all studies reviewed, non-verbal subjects acquired an ordinal rule with one set or series of numerical values and readily apply that rule to novel numerical values without further feedback or training (e.g., Brannon and Terrace, 2000; Brannon, 2002; Cantlon and Brannon, 2006; Picozzi et al., 2010). In other words, infants and animals abstracted ordinal relationships. This generalization of ordinal knowledge reveals that non-verbal organisms appreciate the intrinsic ordinal relationship amongst cardinal values (e.g., 4 < 8 < 12), an ability which goes above and beyond the sequential ordering of numerical sets within the sequence (e.g., 4 → 8 → 12).

Second, non-human animals and infants are less apt to acquire numerical sequences that violate this inherent order, even with repeated trials. This is exemplified in the finding that non-human primates cannot learn to arbitrarily order numerical sets (e.g., 3 → 1 → 4 → 2; Brannon and Terrace, 2000) and infants do not detect inverse number-line length ordinal interrelations (e.g., small numerical sets paired with long lines; de Hevia and Spelke, 2010). This suggests that non-verbal subjects rely upon the “less than” and “greater than” relationships inherent to numerical sequences when abstracting numerical ordinality. In sum, evidence strongly suggests that responding across these tasks was not the result of arbitrary sequence learning, but a function of the numerical values presented.

Interestingly, these findings can be juxtaposed against experiments that show that non-human animals and human infants detect serial order; that is, they readily learn arbitrary orderings of non-quantitative things. For example, non-human animals learn to select the yellow box followed by the blue box followed by the green box, etc. (Gillan, 1981; Boysen et al., 1993; Terrace, 1993; Beran et al., 2004; Merritt et al., 2007). Even infants as young as 4-months of age detect changes in the serial order of moving and sounding objects (Gulya et al., 1998; Lewkowicz, 2004; Lewkowicz and Berent, 2009)^13^. Furthermore, children and non-human animals learn non-monotonic quantitative serial orderings (e.g., medium box → medium-small box → large box → small box → medium-large box) more poorly than monotonic ones (Terrace and McGonigle, 1994; Ohshiba, 1997; Kundey et al., 2010). The intrinsic order of numerical quantities is salient. Although both infants and animals are capable of learning arbitrary sequences, they are less apt (or possibly unable) to do so when the sequences violate numerical ordering. This pattern not only speaks to the shared evolutionary basis of ordinal understanding about number and quantities, but also tells us that numerical order holds a privileged status above mere sequence learning.

## Discussion

From free-ranging dogs deciding whether to retreat from a rival pack (Bonanni et al., 2011) to young human infants reaching for the most bits of cereal (Feigenson et al., 2002), tasks requiring ordination of quantities provide a wealth of evidence showing that both preverbal human infants and non-human animals are sensitive to ordinal relationships. Single-trial experiments assessing spontaneous ordination reveal a robust set size signature in both non-human animals (but, see, Krusche et al., 2010) and preverbal infants. On the other hand, when non-human animals are given repeated attempts to order quantities, set size limitations diminish, and analog-magnitude representations play a primary role. As such, non-verbal understanding about number-related order depends upon both object-based individuation and analog-magnitude systems. Whether a similar pattern holds for repeated trials in RQJ tasks with infants remains an open question ripe for investigation.

Given that number is naturally confounded with other quantitative variables in RQJ tasks, these types of experiments cannot distinguish whether this non-linguistic sensitivity to ordinality is based upon number, surface area, volume, hedonic value, and/or a combination of these quantities. Our examination of numeric appreciations of ordinality (i.e., when non-numerical quantities like surface area, contour length, inter-element distance, and density are prevented from systematically covarying with the cardinality of sets) reveals less similarity between infants and non-human animals. When sequential responses to numerical order are reinforced, the representations that non-human animals form are not limited by set size, but are ratio-dependent (Brannon and Terrace, 1998, 2000; Smith et al., 2003; Judge et al., 2005; Brannon et al., 2006; Cantlon and Brannon, 2006; Scarf et al., 2011).

In contrast, the ability of young human infants (under 9 months) to detect changes in numerical order primarily continues to reveal a set size signature. It is not until the end of the first year of life (∼11 months) that infants reliably detect changes in numerical order regardless of the size of the sets involved (Brannon, 2002). Thus, evidence suggests that the ability to integrate numerical representations generated from the exact (object individuation) and approximate (analog-magnitude) systems increases across development in human infancy. More work is needed to clarify this discrepant pattern of findings across infants and non-human animals, though. Specifically, whether methodological differences (employing associative learning vs. passive observation) may account for these observed differences remains to be determined. That associative learning activates a continuous, broadly applicable system for understanding small and large numerical values (i.e., analog-magnitudes) is a compelling idea. For one, it could inform the creation of educational tools and efforts designed to stimulate an infant’s understanding of number. Further, more work is needed to determine whether the developmental trajectory, an increased ability to integrate numerical representations generated from the exact and approximate systems, is mirrored in the lives of non-human animals. Discovering that the cognitive development of non-human animals follows the trajectory shown by human infants would provide strong evidence to support a shared evolutionary basis of numerical cognition.

Even after our review, many questions remain about the evolutionary origins and adaptive significance of ordinal knowledge and its relation to other cognitive abilities. Surprisingly, although human infants appear capable of detecting ordinal relationships across small and large sets by around 11 months of age (Brannon, 2002), they continue to fail to discriminate changes in set cardinality crossing the small/large set size boundary as late as 23 months of age (i.e., they fail to discriminate a set of two from four; Barner et al., 2007). What is it about ordinality that allows infants to overcome the set size limitations imposed by the employment of two distinct representational systems? One way to shed light on this question is to evaluate the role of individual differences (in terms of plasticity and stability over time) in the development of ordinal abilities. For example, early understandings of numerical order may be correlated with, or predicative of, greater precision in analog-magnitude representations, an ability to detect all kinds of order in the world, and/or mathematical skills. Future research should explore these possibilities. Similar questions may also be addressed with non-human species to shed light on when and why such abilities have emerged and if they are immune to set size limitations early in development.

In conclusion, evaluating the shared basis of ordinal numerical knowledge helps us construct a complete picture about the development and evolution of numerical cognition. Although much has been learned about the signatures of numerical ordinal behavior in both human infants and non-human animals, open questions remain. A stronger parallel between infant and animal paradigms will provide greater insight into the developmental and evolutionary origins of these sophisticated abilities. Ordinal knowledge about number is an evolutionarily ancient, developmentally early, non-linguistic capacity that spans a species’ social system and ecological niche. Because developmental psychology is about comparison and comparative psychology is about development, our fields must continue to track age-related changes in the way that all species understand numerical order.

## Conflict of Interest Statement

The authors declare that the research was conducted in the absence of any commercial or financial relationships that could be construed as a potential conflict of interest.

## Footnotes

^1^There is another aspect of ordinality in which the quantitative attribute defining order is time, not cardinality. Serial order involves the position or place of a single thing within a sequence. For example, one would say that the black horse crossed the finish line first, the brown horse second, and the white horse third. Indeed, first, second, third, and so forth are called ordinal numbers in mathematics.

^2^For an order relation to exist, four properties must stand (Russell, 1903/1996; Stevens, 1951): (1) irreflexivity (*a* ≠ *b*, *a* ≠ *c*, etc.), which means that no term is related to itself; (2) asymmetry, which means that a series is unidirectional (if *a* → *b* then it does not hold that *b* → *a*); (3) connectedness, which indicates that the relation holds between all pairs in the series (if *a* → *b* → *c* → *d* then *a* → *b*, *a* → *c*, *a* → *d*, *b* → *c*, *b* → *d*, and *c* → *d*); and (4) transitivity, which relates to the relationship that exists between trios in the series (if *b* → *c* and *c* → *d* then *b* → *d*).

^3^Note that we reviewed only evidence gathered from non-language-trained animals. We did so because researchers theorize that language promotes a kind of flexible thinking that leads to the development of higher levels of conceptualization (Kotovsky and Gentner, 1996; Hauser and Carey, 1998; Fabre-Thorpe, 2001).

^4^The data from one report (Irie-Sugimoto et al., 2009) suggest that Asian elephants possess either an exact representation system capable of holding up to six items or a relatively precise analog-magnitude system that is similar to adult humans (e.g., 7:8 ratio; Halberda and Feigenson, 2008).

^5^Ibid footnote 4, p. 8.

^6^Beran (2012) also reported that the chimpanzee continued to make the ordinal response when one set in the pair was made visible, which resulted in the ratio-dependency disappearing. This finding is consistent with the claim that the object individuation system is used only for representing visual sets (vanMarle and Wynn, 2009).

^7^Lion prides were more likely to approach the location of playback roars from a single rather than three intruders regardless of the number of defending adults in their own pride (McComb et al., 1994). Because no other numerical comparisons were tested, though, it is impossible to ascertain whether the data are consistent with an analog-magnitude or object-based individuation mode of representation.

^8^Hedonic value refers to the idea that differences in affective and appetitive responses account for subjects responding in an ordinal manner. For example, choosing and receiving the largest quantity when presented with two vs. three food items induces more salivation and satiation and elicits a stronger affective response than choosing and receiving the smaller quantity.

^9^Compare this to assessments of serial order. Subjects respond or are habituated to arbitrary stimuli in an experimenter-specified sequential order (if the letters denote different colored boxes, Y → B → G → R → O). Alternatively, in the transitive inference paradigm, subjects are trained to select the positive stimulus in a pair across an overlapping series of stimuli (Y+/B−, B+/G−, G+/R−, and R+/O−).

^10^Evidence from Suanda et al. (2008) indicates that 9-month-olds detect ordinal direction changes in sequences that have multiple redundant cues (i.e., when cumulative item area, individual item size, *and* the number of items were positively correlated within sequences), but not when number is the sole cue to ordinal direction.

^11^There is a large body of evidence showing that non-human animals can be trained to order symbolic representatives of quantity (Boysen et al., 1993; Boysen and Berntson, 1995; Harris et al., 2007, 2010; Beran et al., 2008c). For the purposes of this review, however, we focus on appreciations of number-related ordinal relationships that are independent of linguistic or symbolic systems.

^12^These tasks followed training to touch the full sequence in ascending (e.g., 1 → 2 → 3 → 4) or descending order (e.g., 4 → 3 → 2 → 1).

^13^Ibid footnote 9, p. 9.
